# Application of carbon nanoparticles in laparoscopic sentinel lymph node detection in patients with early-stage cervical cancer

**DOI:** 10.1371/journal.pone.0183834

**Published:** 2017-09-05

**Authors:** Yan Lu, Jin-Ying Wei, De-Sheng Yao, Zhong-Mian Pan, Yao Yao

**Affiliations:** Department of Gynecologic Oncology, Affiliated Tumor Hospital of Guang Xi Medical University, Nanning, People’s Republic of China; Istituto di Ricovero e Cura a Carattere Scientifico Centro di Riferimento Oncologico della Basilicata, ITALY

## Abstract

**Objective:**

To investigate the value of carbon nanoparticles in identifying sentinel lymph nodes in early-stage cervical cancer.

**Methods:**

From January 2014 to January 2016, 40 patients with cervical cancer stage IA2–IIA, based on the International Federation of Gynecology and Obstetrics (FIGO) 2009 criteria, were included in this study. The normal cervix around the tumor was injected with a total of 1 mL of carbon nanoparticles (CNP)at 3 and 9 o'clock. All patients then underwent laparoscopic pelvic lymph node dissection and radical hysterectomy. The black-dyed sentinel lymph nodes were removed for routine pathological examination and immunohistochemical staining.

**Results:**

Among the 40 patients, 38 patients had at least one sentinel lymph node (SLN). The detection rate was 95% (38/40). One hundred seventy-three SLNs were detected with an average of 3.9 SLNs per side. 25 positive lymph nodes, which included 21 positive SLNs, were detected in 8 (20%) patients. Sentinel lymph nodes were localized in the obturator (47.97%), internal lilac (13.87%), external lilac (26.59%), parametrial (1.16%), and common iliac (8.67%) regions. The sensitivity of the SLN detection was 100% (5/5), the accuracy was 97.37% (37/38), and the negative predictive value was 100. 0% and the false negative rate was 0%.

**Conclusions:**

Sentinel lymph nodes can be used to accurately predict the pathological state of pelvic lymph nodes in early cervical cancer. The detection rates and accuracy of sentinel lymph node were high. Carbon nanoparticles can be used to trace the sentinel lymph node in early cervical cancer.

## Introduction

Sentinel lymph node (SLN) mapping is a viable surgical strategy determine whether to perform radical lymphadenectomy. It is based on the idea that a SLN is the first stop in lymph node metastasis. If a SLN does not metastasize, then theoretically the lymph nodes after the SLN should also be negative. The postoperative pathology-confirmed lymph node metastasis rate of most cervical cancer patients is approximately 24.16% [1]. They do not benefit from pelvic lymph node (PLN) dissection. The intention of SLN biopsy is to avoid overtreating by performing a complete PLN dissection of negative lymph nodes. This approach can avoid radical lymphadenectomy complications such as nerve injury, lymphatic edema, and lymphatic cyst [2], which can seriously affect a patient’s quality of life. SLN mapping achieves individualized treatment of cancer patients.

SLN mapping may be an acceptable surgical strategy for radical lymphadenectomy in patients with early-stage cervical cancer and it has been used successfully in treating breast cancer or skin melanoma. Although SLN mapping of cervical cancer is still in its infancy, SLN biopsy for the early diagnosis of cervical lymph node metastasis may be reliable and feasible. Carbon nanoparticles (CNP) consist of ordinary activated carbon transformed through a technical treatment into very small particles. Smooth carbon particles with a diameter of approximately 21 nm are added in the form of an average particle size of 150 nm, after adding a suspension of polyvinylpyrrolidone and physiological saline. The capillary endothelial cell gap is 30–50 nm and the capillary lymphatic endothelial cell gap is 100–500 nm. Therefore, CNP can quickly enter lymphatic vessels and lymphatic capillaries through macrophage phagocytic action and remain in the lymph nodes. The CNP do not enter the blood vessels and only enter the lymphatic capillaries. It is easy for surgeon to distinguish black lymph nodes, which contrasts with the surrounding. CNP, a type of biological dye with good lymphatic tendency, is a popular SLN tracer in mapping SLN of cervical cancer.

The aim of our study was to assess the accuracy of laparoscopic SLN biopsy performed with cervical CNP navigation. The aim of this study was to evaluate the SLN detection rate, specificity, sensitivity, and false-negative rate, with regard to the routine pathological results of complete lymphadenectomy. We also evaluated the optimum time from CNP injection to mapping.

## Materials and methods

### Ethics statement

This study has been approved by the Medical Ethics Committee of Guang Xi Medical University Affiliated Tumor Hospital. All patient who was enrolled in this study has signed the informed consent.

General material from January 2014 to January 2016, 40 patients with early–stage cervical cancer with International Federation of Gynecology and Obstetrics (FIGO) stage IA2–IIA disease underwent laparoscopic SLN mapping at the Department of Gynecologic Oncology, Affiliated Tumor Hospital of Guang Xi Medical University (Nanning, China). All patients were underwent laparoscopic SLN mapping with radical hysterectomy and systemic pelvic lymphadenectomy with or without para-aortic lymphadenectomy.

The exclusion criteria were as follows: (1) patients who had undergone cervical treatment such as cervical loop electrosurgical excision procedure, and conical resection; (2) patients whose imaging findings showed positive retroperitoneal lymph nodes, distant metastases, or previous retroperitoneal surgery; (3) patients who had undergone preoperative radiotherapy; (4) patients with severe abdominal adhesions. The following items were collected: median patient age, median body mass index (BMI), and histologic type and grading. Estimated median blood loss, median laparoscopy time, and intraoperative and postoperative complications were analyzed intraoperatively (Table 1).

**Table 1:** The patients’ demographic and clinicopathologic information.

**Demographic and clinical information (n = 40)**		
**Median age, years (range)**	42	(34–53)
**Median BMI, kg/m** ^**2**^ **(range)**	26	(18.9–52)
	N	(%)
**Clinical stage**		
IA2	2	5.00%
IB1	23	57.50%
IB2	9	22.50%
IIA1	4	10.00%
IIA2	2	5.00%
**Histology**		
Squamous cell carcinoma	30	75%
Adenocarcinoma	7	17.50%
Adenosquamous carcinoma	3	7.50%
**Tumor size (cm)**		
Microscopic	2	5.00%
≥1 and 2 cm	6	15.00%
≥2 and 3 cm	8	20.00%
≥3 and 4 cm	13	32.50%
≥4 cm	11	27.50%
**Histological grade**		
G1	11	27.50%
G2	16	40.00%
G3	12	30.00%
NA	1	2.50%
**LVSI**		
Yes	18	45.00%
No	22	55.00%
**Neoadjuvant chemotherapy**		
(with paclitaxel and oxaliplatin, 1–2 courses)		
Yes	11	27.50%
No	29	72.50%
**HPV type**		
HPV-16	26	65.00%
HPV-18	4	10.00%
Coinfection	10	25.00%

BMI, body mass index; HPV, human papilloma virus; LVSI, lymphovascular space invasion.

Pretreatment evaluation consisted of medical history, physical examination, and computed tomography(CT) scan. Magnetic resonance imaging (MRI) of the pelvis was used to rule out parametrial invasion.

### The SLN biopsy technique

Before surgery, the patient was placed in the lithotomy position. The CNP method starts with injection after anesthesia induction and before placing the trocars. At the noted time, The normal cervix around the tumor was injected with a total of 1 mL of carbon nanoparticles (CNP) at 3 and 9 o'clock, with deep (0.3–0.5cm) cervical injection.The process of injection lasted at least 3 minutes, and then some pressure was applied locally to prevent CNP extravasation after the injection. The first black-stained lymph nodes appearing after initiating the injection were identified as the SLNs. The black-stained lymphatic vessels were observed under the laparoscope. After identifying the black-stained lymph nodes as the SLNs, the number and location of SLNs were recorded (If the mapping time exceeds 10 minutes, we do not define the black-stained lymph nodes as SLNs). They were then removed and sent to the pathological examination alone after resection. After obtaining the SLN, all patients underwent laparoscopic radical hysterectomy and pelvic lymph node dissection (and/or para-aortic lymph node sampling). The same surgeon performed all surgical procedures.

### Pathological examination

A group of experts of gynecologic oncology pathologist, who was highly skilled in the analysis of SLNs, examined all surgical samples and SLNs. The pathologic criteria for successful SLN staining were CNP in the lymph nodes. The SLNs and non-SLNs were handled in the standardized manner. All lymph nodes with macroscopic metastases were sectioned. The SLNs that appeared normal were cut perpendicular to the long axis. Definitive histology by paraffin embedding and serial step sectioning was thereafter performed. Slides (5 micrometers) were stained with hematoxylin and eosin, followed by routine immunohistochemical staining with anti-Pan cytokeratin. The non-SLNs were similarly processed, and were only measured for routine hematoxylin and eosin levels.

## Statistical analysis

### Consistency of SLN and PLN metastasis using the kappa coefficient test

The detection rate was calculated as the number of patients with at least one identified SLN divided by the total number of enrolled patients. The accuracy of SLN mapping was determined by calculating the sensitivity, specificity, and positive and negative predictive values Our statistical method is used X±S for measurement data.The Fisher exact was used for comparing the correlation between tumor size and the SLN detection rate. Statistical analyses were performed using SPSS statistical software, version 17.0. (SPSS Inc., Chicago, IL,USA)

## Results

### The time to the first appearance of black-stained lymph nodes and the rate of black staining

The shortest time before the appearance of black-stained lymph nodes was 4 minutes and the longest time was 10 minutes (The time for carbon nanoparticle staining of SLN is no more than 10 minutes, it can minimize the unfavorable staining of non-SLNs). The mean time was 8.5 ± 1.5 minutes. The rate of black staining was 14.89% (173/1162). (Table 2)

**Table 2:** The staining time of SLNs.

Period(min)	Number	%
≥4 and 5	1	2.5
≥5 and 6	1	2.5
≥6 and 7	2	5.0
≥7 and 8	6	15
≥8 and 9	12	30
≥9 and ≤10	18	45

### The detection rate and distribution of the SLNs

**Fig 1:**
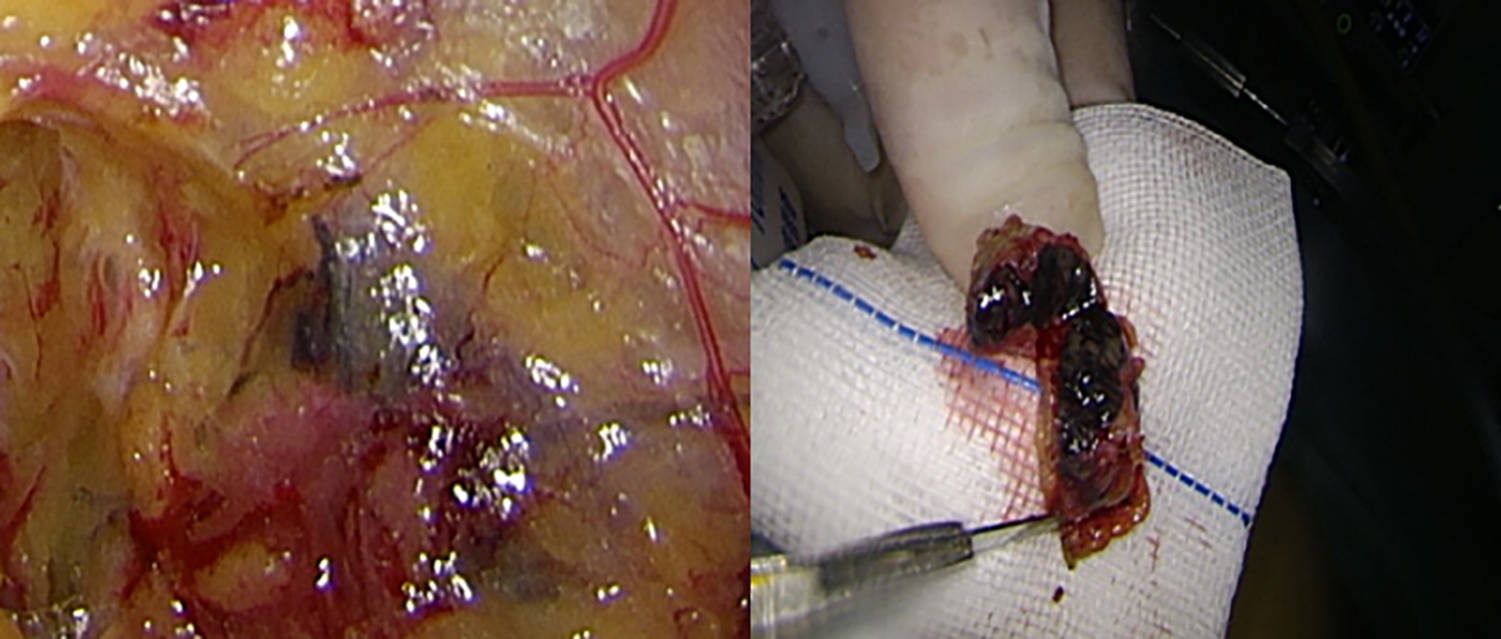
Right external iliac sentinel lymph node. (A) Intraoperative right external iliac lymph node mapping. (B) Postoperative right external iliac lymph node profile.

**Fig 2:**
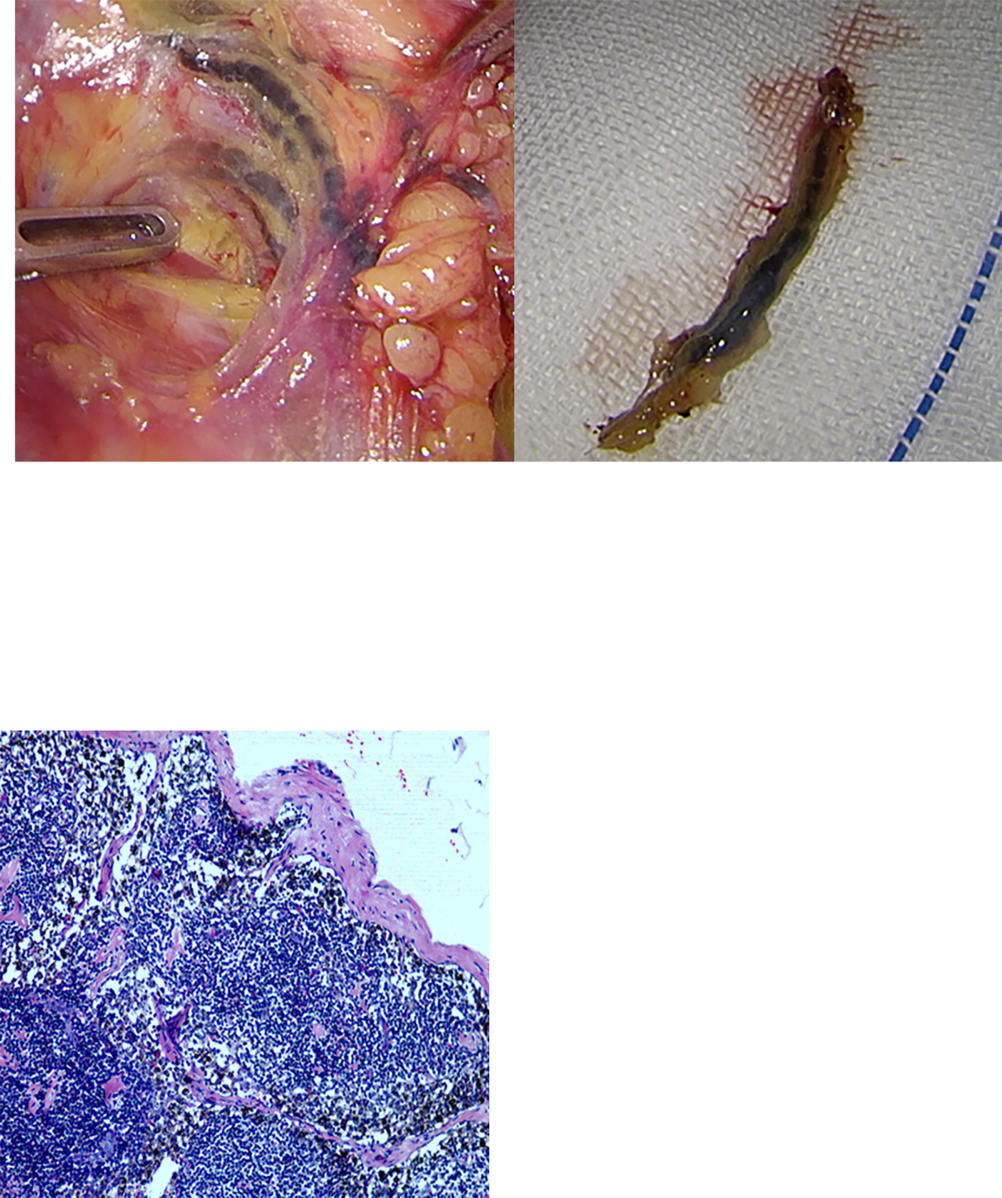
Black-stained lymphatic vessels. (A)Retroperitoneal black stained lymphatic vessels. (B)CNP black stained lymphatic vessels.

**Fig 3:**
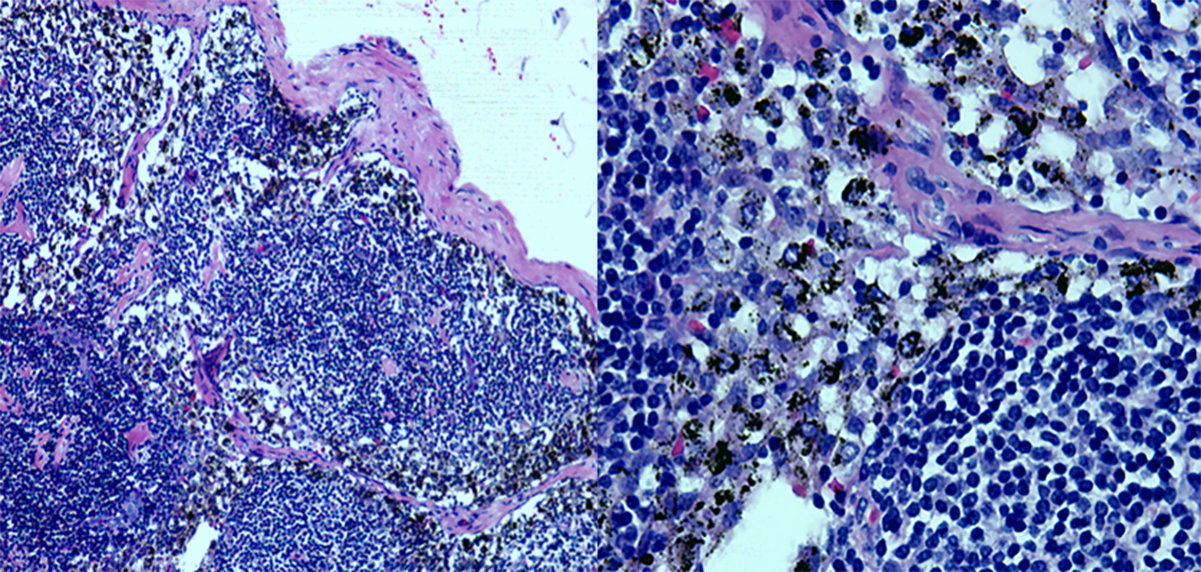
Black-stained sentinel lymph node. (A)SLN of hematoxylin-eosin(HE)stained pathology(100x). (B)SLN of hematoxylin-eosin(HE)stained pathology(400x).

Among the 40 patients, 38 patients had at least one SLNs. In these patients, the detection rate of SLN was 95% (38/40). SLNs were detected with preoperative chemotherapy in 9 of 11 patientsand the detection rate was 81.82%. SLN mapping was obtained in 20 patients for a bilateral detection rate of 52.63%, and unilateral SLNs detection was obtained in 18 patients (47.37%). Among 18 patients with unilateral SLNs, there were 28 SLNs were on the left side and 30 SLNs were on the right side. A total of 1162 PLNs were removed from 40 patients. The average number removed was 27 PLNs for each patient. The total number of SLNs resection was 173 nodes, accounting for 14.89% of total PLNs (173/1162). The average number of SLN detected was 3.9 (range, 1–6 nodes) in each patient, which were distributed in the obturator, internal iliac, external iliac, common iliac, and para-aortic regions (Figs 1–3). Of these 38 patients, SLNs were detected in 29 patients with a tumor less than 4 cm (i.e., a detection rate of 100% [29/29]), whereas SLNs were not detected in two patients with tumors larger than 4 cm (i.e., a detection rate of 81% [9/11]).

### The distribution of PLNs and the SLNs in the pelvis

In pelvic lymphadenectomy, there were two lymph nodes appearing in the para-aortic and parametrial tissue, respectively, and one in the sacrum. The PLNs were most frequently in the obturator (37.52%), external iliac (29.52%), internal iliac (13.94%), common iliac (12.13%) and para-aortic (1.8%), parametrial (5.09%) and sacral (0%) regions (Table 3). Among the 40 patients, 8 (20%) patients had 25 lymph nodes metastases, which included 21 SLNs and 4 non-SLNs. Of the 38 patients in whom SLNs had been successfully identified, 6 patients had SLN metastases. 5 patients had SLN and PLN metastasis. 1 patients had SLNs metastasis but no PLN metastases. No patient had just PLN metastases and no SLN metastasis. In addition, 32 patients had no metastasis in the SLNs and PLNs (Table 4). The sensitivity was 100%. The specificity of the prediction was 96.97%. The accuracy was 97.37%. The negative predictive value was 100% and the false positive rate was 0%. The kappa coefficient was 0.894, which indicated that SLN and PLN metastasis had a high degree of consistency (Table 5). All patients with tumor diameter was ≤4 cm were found SLNs. While the tumor diameter was >4 cm, there were only 9 patients could be detected SLNs and 2 patients couldn't be detected SLNs, but the difference was not statistically significant (*P>0*.*05*). (Table 6). However, we found the SLN detection rate was 100% (8/8) when the tumor diameter ≤2cm, but was 93.75% (30/32) when the diameter was >2 cm, and the difference was statistically significant (*P<0*.*05*). (Table 7).

**Table 3:** The distribution of PLNs and SLNs.

Location	Total number of PLNs (%)	Total number of SLNs(%)
**Obturator**	436 (37.52%)	83 (47.97%)
**Internal iliac**	162 (13.94%)	24 (13.87%)
**External iliac**	343 (29.52%)	46 (26.59%)
**Common iliac**	141 (12.13%)	15 (8.67%)
**Para-aortic**	21 (1.80%)	2 (1.16%)
**Parametrial**	59 (5.09%)	2 (1.16%)
**Sacral**	0 (0%)	1 (0.58%)
**Total**	1162 (100%)	173 (100)

PLN, pelvic lymph node; SLN, sentinel lymph node.

**Table 4:** The distribution of positiveSLNs.

Location	N	%
**Obturator**	9	42.86
**External iliac**	5	23.81
**Internal iliac**	4	19.05
**Parametrial**	3	14.28
**Total**	21	100

**Table 5:** The pathologic results of PLNs and SLNs.

SLN	PLN		Total
	+	-	
**+**	5	1	6
**-**	0	32	32
**Total**	5	33	38

**Table 6:** The tumor size and the sentinel lymph node detection rate.

Tumor size	SLN detection	Non-SLN detection	Total
**≤4cm**	29	0	29
**>4cm**	9	2	11
**Total**	38	2	40

*P* = 0.071 based on the Fisher exact used to compare the correlation between tumor size and the SLN detection rate.

**Table 7:** The tumor size and the sentinel lymph node detection rate.

Tumor size	SLN detection	Non-SLN detection	Total
**≤2cm**	8	0	8
**>2cm**	30	2	32
**Total**	38	2	40

*P* = 0.000 based on the Fisher exact used to compare the correlation between tumor size and the SLN detection rate.

## Discussion

Performing a SLN biopsy under laparoscopic surgery in an early malignant tumor of uterus was feasible. The sensitivity of SLN detection is 92% and the negative predictive value is as high as 98.2%, based on the findings of many studies [3]. Lymph node involvement remains the most important prognostic factor for women with cervical cancer. Early stage cervical cancer fortunately has a low risk of lymph node metastasis. The incidence of lymph node metastasis is 1%–16% in patients who have tumors <2 cm, but the incidence increases to 15%–31% in patients who have tumors >2 cm. This finding demonstrates that most patients do not benefit from pelvic and aortic lymphadenectomy [4]. Laparoscopic SLN biopsy is an acceptable and valid approach for patients with apparently confined early-stage cervical cancer, which allows lower surgical morbidities such as lymphedema (10%–15%), lymphocyst (20%), neurovascular injury, ureteral injury, vein injury [5]. It may be very important to integrate SLN biopsy into the management of early-stage cervical cancer. It is a safe and feasible procedure to reduce the parametric resection for patients with negative SLNs and a small tumor load [6]. Recent research has shown that the SLN detection rate is 97.5% (with a bilateral detection rate of 73.3%) with no false negatives or bad reaction [7].

The clinical value of SLN mapping is based on high sensitivity and reliable negative predictive value to predict nodal involvement. Since the preliminary experience of Cibula et al. [8] was published, the most widely investigated tracers for SLN mapping are technetium-99m radiocolloid (99mTc), blue dye (methylene or isosulfan blue), and combination method. The sensibility of the three methods are 81%[9], 91% [10], and 97% [11], respectively. Fluorescent indocyanine green (ICG) dye has been used in SLN mapping in various solid tumors. ICG has recently shown promising results in gynecologic oncology [12, 13], which can improve the detection rate of bilateral lymph nodes through fluorescence imaging [14]. However, there are many literatures have reported that ICG needs to use a special fluorescence laparoscopy, and it can easily spill from the lymphatic when resection lymph nodes because of its small size. It is not easily for surgeon to distinguish SLN from non-SLN if there is serious surrounding green exosmosis and then lead to the false negative rate increased. There is a multicenter trial investigated by Japan Clinical Oncology Group demonstrated that the proportion of false negatives was too high to use ICG to show SLNs [15].

CNP has shown promising results in gynecologic oncology with advantages of small particles, lasting development, and fast dispersion. Leong et al. [16] have reported much experience with using CNP as a mapping technique for SLN biopsy in thyroid papillary carcinoma with false-negative rates of 5.2% and detection rates of 93.3%. The detection rate of CNP is high, but false-negative rates still occur. The reasons maybe because of a large tumor, tumor embolus formation, great-leap metastasis, and different operation techniques used by the personnel. These factors would disrupt the identification of SLN. The false-negative rate of our research was 0%, which may be because of the small sample (40 patients) used in our study.

In our series, CNP-labeled SLNs seem to be feasible in the setting of laparoscopic hysterectomy and PLN dissection with an detection sensitivity of 100% (4/4), predictive specificity of 94.12% (32/34), accuracy of 94.74% (36/38), negative predictive value of 100.0% and false-negative rate of 0%, which verifies that CNP has more advantages than traditional tracers. In the preliminary experience of Liu et al. [17], at least one SLN was successfully detected in 20 (95.24%) patients and 158 SLNs were detected. The conventional pathology results suggested that five (23.81%) patients had positive lymph nodes (n = 16, including 4 in 14 patients). The new approach for SLN detection showed a sensitivity of 80.0% (4/5), accuracy of 100.0% (20/20), and negative predictive value of 100.0% (16/16). Du et al. [18] reported that the sensitivity of SLN detection was 100%, the accuracy of diagnosing lymph node metastasis was 100%, the false-negative rate was 0%, the negative predictive value was 100% among the IA2-IB1 cervical cancer patients.

Our study also showed a SLN detection rate of 12.13% and 1.80% in the common iliac region and para-aortic region, respectively. In particular, common iliac SLNs are often detected between the psoas muscle and common iliac artery and vein. These areas are often overlooked, which suggests that CNP-stained lymph nodes are easy for a surgeon to identify and surgically remove.

We think the reason for tracer failure are as follows. First, on injecting carbon nanotubes injected into a tumor or blood vessels, the background will be concentrated and reduce the detection rate of SLNs. Second, if the tumor is very large, tumor central necrosis will occur and lead to CNP retrograde leakage through the cervical canal to the vagina. Third, preoperative radiotherapy and chemotherapy may affect lymphatic drainage so that actual SLNs cannot be positioned, which results in an increased false-negative rate and reduces the detection rate of SLN. Fourth, tumor size is another factor that affects SLN mapping. Zarganis et al. [19] and Wydra et al. [20] found that the SLN detection rate was 94.1% and 95.6%, respectively, when the tumor diameter was <2 cm, but 78.2% and 58%, respectively, when the tumor diameter was >2 cm. In this study, we analyzed that the tumor diameter >4 cm and ≤ 4cm on the detection rate, the difference was not statistically significant. However, while the tumor diameter is ≤ 2cm, the SLN detection rate was significantly increased, it means tumor diameter is correlated with the detection rate of SLN. The detection rate is the highest. When the tumor diameter is ≤ 2 cm. If a tumor is very large, the vaginal vault is also feasible for the administration of CNP injection because the transfer approach of the upper vaginal wall and cervical cancer is in the same direction [21].

In 1979, Buchsbaum described cervical lymph node metastasis in three ways, and this classic transfer pathway has been widely recognized. The SLNs were most commonly identified in the external iliac basins in current report [22].Ouldamer et al. [23] summarized 27 literature reports of 1301 cases of early cervical cancer and found that the most common SLNs are obturator, internal iliac, and external iliac lymph nodes, which accounted for 83.7% of nodes, whereas SLNs were rare in the common iliac region (6.6%), periaortic region (2%), and the groin area (0.07%).

The parametrial lymph nodes are the first stop in drainage, but they are often neglected by pathologists and gynecologic oncologists. Therefore, data on parametrial lymph node is not well documented. In our study, the most common SLNs were obturator (47.97%), external iliac (26.59%), internal iliac (13.87%), and common iliac (8.67%) regions. Positive lymph nodes were in the obturator (42.86%), the external iliac (23.81%), the internal iliac (19.05%), and parametrial (14.28%) regions. The positive lymph nodes were highly consistent with the distribution of the pelvic lymph nodes.

We found that the proportion of parametrial lymph node was 5.09%, but the detection rate of the SLN of parametrial lymph node was 1.16%; the positive rate of SLN lymph node detection was 14.28%. The current detection rate of parametrial lymph nodes is not high. This finding may be because CNP stains the surrounding tissue black led to local tissue tracer height concentration, which affects the detection of parametrial lymph nodes. In addition, the overlook of clinicians and pathologists is also affecting the detection of parametrial lymph nodes, the active participation of gynecologic oncologists can help solve this problem. Benedetti-Panici et al. [24] reported that the parametrial lymph node detection rate was 59%–93%, the number of parametrial lymph nodes detected per patient was 2–5, and in up to 1 sentinel node was detected among 25 parametrial lymph nodes. In this study, a considerable proportion of patients failed to detect parametrial lymph nodes. The possible reasons are that parametrial lymph nodes are smaller and buried deeply in the uterine tissue, and the black-stained cervix make identifying parametrial lymph nodes more difficult, which affects the detection rate. For these reasons, the current cervical lymph node drainage is controversial. This study involved a limited number of cases. Therefore, further large sample studies are needed.

The application of CNP in the laparoscopic detection of SLNs is a feasible method with a high SLN detection rate and ideal false-negative rate. It can be used to accurately predict PLN metastases in patients with early cervical cancer. The application of CNP in gynecological tumors is not frequent. Many issues need to be resolved such as SLN imaging instability (i.e., which tracers have a higher sensitivity), the appropriate tracer dose and concentration, and how the negative rate can be accepted. It cannot replace comprehensive pelvic lymph node dissection in cervical cancer. However, there is promise that it can reduce surgical complications and improve the quality of life of patients. It is worthy of further research technology, and multicenter large sample studies are needed.
